# Body mass index and quality of life in individuals with polycystic ovary syndrome: Dysmorphic concerns and eating disorders as mediators

**DOI:** 10.3389/fpubh.2022.962083

**Published:** 2022-10-05

**Authors:** Nadia Barberis, Danilo Calaresi, Marco Cannavò, Valeria Verrastro

**Affiliations:** ^1^Department of Health Sciences, Magna Græcia University, Catanzaro, Italy; ^2^Department of Clinical and Experimental Medicine, University of Messina, Messina, Italy

**Keywords:** body mass index, dysmorphic concerns, eating disorders, health-related quality of life, polycystic ovary syndrome

## Abstract

**Objective:**

Weight issues, dysmorphic concerns and eating disorders are common among individuals with polycystic ovary syndrome (PCOS) and are linked to poor quality of life (QoL). The goal of the current study was to examine whether the association between body mass index (BMI) and QoL was mediated by dysmorphic concerns, examining also the relations with eating disorder symptomatology.

**Methods:**

Questionnaires were administered to 435 individuals with PCOS aged between 18 and 40 years (*M* = 27.62; *SD* = 4.83) to measure dysmorphic concerns, eating disorder symptoms and QoL. Participants were also asked to report their height and weight to compute their BMI.

**Results:**

Structural equation modeling was used to observe the relationship between the variables. The results revealed a direct relationship between QoL, BMI, dysmorphic concerns and eating disorder symptomatology. In addition, dysmorphic concerns were related to BMI and eating disorder symptomatology. Furthermore, the results showed the mediating role of dysmorphic concerns and eating disorder symptoms in the relationship between BMI and QoL.

**Conclusion:**

The current results highlight the potential importance of harmful relationships with one's own body and food, explaining why weight issues may be linked to different levels of QoL in PCOS individuals. The implications of these findings are discussed.

## Polycystic ovary syndrome

Polycystic ovary syndrome (PCOS) is a disabling endocrinopathy affecting approximately 6–10% of women of childbearing age (1). The condition is multifactorial and often difficult to diagnose due to the existence of different diagnostic criteria (2), mainly based on the presence of a combination of clinical signs of anovulation or menstrual irregularities, polycystic ovaries and androgen excess (3). Weight management issues exacerbate the clinical significance of the disease, leading to important clinical concerns such as diabetes and cardiovascular disease (4, 5). Not surprisingly, research has shown that weight problems are among the most burdening symptoms of this condition, whereas others, such as infertility or menstrual irregularity, have a moderately negative impact on individuals' quality of life (QoL) (6).

Past findings showed how patients with PCOS are more likely to be overweight compared to controls and that PCOS is common in obese women (7, 8). Likewise, previous research has found that the prevalence of PCOS in women with weight problems is about 25% and it is widespread in patients undergoing bariatric surgery (9, 10). The research emphasized how a problematic body mass index (BMI) may foster a negative self-view (11–13) and may negatively influence one's own perceived body image. For instance, past findings showed how BMI is related to dissatisfaction with one's own body in both normal (14–17) and clinical populations (12, 18); furthermore, longitudinal research (19) revealed that BMI was a risk factor for decreased body satisfaction at the time of initial assessment and 5 years later.

Adherence to a healthy lifestyle, namely, proper nutrition and adequate physical activity, is therefore an important part of optimal PCOS management (20). Thus, lifestyle interventions focused on weight and diet to reduce symptoms and improve health outcomes have been indicated as first-line treatment for individuals with PCOS (20). However, previous research observing these kinds of treatments suggested that they provide small or short-term effects (21). It is thus necessary to identify alternative target areas to improve their clinical efficacy.

Given that people with PCOS who have a high BMI are more likely to report poor health status (22, 23) and that having a problematic body weight can lead to fear of social evaluation and perceptions of flaws in appearance (24–26), it is reasonable to assume that dysmorphic concerns are likely to occur.

### The interplay between dysmorphic concerns and eating disorder symptomatology in PCOS

Dysmorphic concerns refer to perceived aesthetic flaws and the use of strategies to disguise them, withdrawal from social events due to appearance concerns and also reassurance-seeking about one's own appearance from significant others (27). While body dissatisfaction refers to a negative subjective evaluation of a person's physical body size and/or shape (28–30), dysmorphic concern is a more specific concept that encapsulates not only behavioral, emotional, and cognitive components related with negative body image (27, 31) but also the presence of maladaptive behaviors aimed at altering one's body (32, 33). One of the components of dysmorphic concerns is in fact the presence of potentially harmful behaviors aimed at altering the body through excessive fasting and physical exercise, performance enhancing substances, engaging in excessive grooming, and undergoing cosmetic surgical treatments (34, 35). As such, dysmorphic concerns entail distorted beliefs that one's body is defective that lead to marked social withdrawal (32, 33) and they seem more recurrent in women (36). Previous findings showed a strong association between PCOS and dysmorphic concerns (37–39), and showed that patients with PCOS have worse dysmorphic concerns than individuals without this condition (37). In particular, concerns about weight have been identified as a major component of self-perceived impairment in patients with PCOS (20). In effect, negative thoughts and feelings, such as those associated with dysmorphic concerns, are closely related to impaired QoL (40). The nature of the relationship between dysmorphic concerns and QoL in individuals with PCOS was examined in a meta-analysis by Bazarganipour et al. (41), who found that QoL impairment was exerted by dysmorphic concerns through a significant link with the physical manifestations of hyperandrogenism. Furthermore, dysmorphic concerns have been correlated to a variety of risk factors for poor QoL in people with PCOS, including appearance evaluation, body area satisfaction, overweight preoccupation and higher self-classified weight (42, 43).

Of note, body image disturbances such as dysmorphic concerns are key components of eating disorders (44, 45). Eating disorders are a set of pathologies based on abnormal food intake and preoccupation with weight that can hinder a person's functioning (46). The burden of eating disorder symptomatology on many spheres of a person's life is well documented, with studies revealing diminished interpersonal functioning (47, 48) and increased morbidity and healthcare costs (49). It is thus reasonable to argue that more severe eating disorder symptoms may be positively related to worsened QoL. A connection between PCOS and eating disorders has been suggested by a few studies (50) and some insights have shown that individuals with PCOS display altered dietary intakes and higher odds of presenting with food cravings (42–45, 47–52). For instance, Bernadett and Szemán (53) underlined that the prevalence of clinical and subclinical bulimia nervosa is higher among individuals with PCOS compared to controls. In addition, those with PCOS appear to be more likely to report food restriction and weight concerns, which are also key factors in eating disorders, compared with healthy individuals (50, 54, 55). The development of disordered eating in individuals with PCOS seems to be significantly linked to distorted perceptions and beliefs about one's own body image (56–58). In particular, body self-evaluation and drive for thinness appear to be among the most prominent components in the maintenance and development of eating disorders, even in individuals with PCOS (59, 60).

### The present study

The above studies underline how interactions between biopsychosocial factors may account for different levels of QoL in individuals with PCOS. There is, in fact, good evidence that body dissatisfaction and/or eating-disordered behavior mediate the association between (elevated) BMI and impairment in quality of life among both women and men in the general population and among both young people and adults (29, 61) and thus is reasonable to infer that said interactions are likely to occur in individuals with PCOS as well. Indeed, it has been highlighted that BMI is a biological dimension that is closely associated with lower body dissatisfaction (62, 63), thereby suggesting that problematic body fatness may constitute a plausible antecedent accounting for individual variation in the preoccupation with one's own physical characteristics (25, 64) and thus dysmorphic concerns. Dysmorphic concerns are in fact a constellation of preoccupations with perceived defects in one's own body that previous findings have shown to foster harmful relationships with food and weight management.

PCOS, as a chronic condition associated with unhealthy BMI and several psychosocial impairments (65–67), is thought to impair one's own QoL. For these reasons, the aim of the current study is to test the hypothesis that an association between BMI and QoL in women with PCOS would be mediated by dysmorphic concerns, examining also the relations with eating disorder symptomatology. Specifically, the goal of this research was to test a model in which higher BMI predicts higher levels of dysmorphic concerns, which in turn lead to higher levels of eating disorder symptomatology that consequently lowers the QoL. It is also expected that higher BMI and dysmorphic concerns predict lower QoL.

## Method

### Participants

Questionnaires were administered to 435 individuals with PCOS aged between 18 and 40 years (M = 27.62; SD = 4.83).

### Measures

#### Quality of life

*Polycystic Ovary Syndrome Health-Related Quality of Life Questionnaire (PCOSQ;* (68)). It is a self-report questionnaire designed to s assess specifically the QoL of individuals with PCOS. The questionnaire consists of 26 items that investigate the impact of the disease on several aspects of one's quality of life (e.g., “Concerned with infertility problems”). Individuals must respond on a seven-point Likert scale (7 = optimal function, 1 = poorest function). High scores indicate good HRQoL. In our study, Cronbach's alpha was 0.91.

#### Eating disorders

*Eating Attitudes Test* [EAT-26; (69)]. It is a 26-item self-report questionnaire designed to assess eating disorders symptomatology (e.g., “I am terrified about being overweight”). Participants are asked to respond using a six-point Likert scale ranging from zero (= “Never”) to five (= “Always”). High scores indicate abnormal eating behaviors. In our study, Cronbach's alpha was 0.88.

#### Dysmorphic concerns

*Italian Body Image Concern Inventory* (I-BICI; (31)). It is a self-report questionnaire that evaluates dysmorphic concerns (e.g.: “I spend a significant amount of time checking my appearance in the mirror”). Participants are asked to respond on a five-point Likert-type scale ranging from one (= “Never”) to five (= “Always”). Higher scores indicate greater dysmorphic concerns. This questionnaire was widely used to assess PCOS patients (70–72) and it was previously used in cross-sectional research in the Italian context to observe dysmorphic concerns (73, 74). In our study, Cronbach's alpha was 0.93.

### Procedures

The protocol was created using an online survey and participants were recruited through social networks by targeted posts on individuals suffering from PCOS in thematic groups from 21 December 2021 to 22 March 2022. Inclusion criteria were: having physician-diagnosed PCOS for at least 1 year, being ≥18 years of age and being able to speak Italian. Participants were considered ineligible if the following were present: lack of a PCOS diagnosis from a health professional, the presence of gynecological or endocrinological comorbidities, pregnant at the moment of protocol administration, insufficient fluency in Italian or < 18 years of age.

All participants declared that they voluntarily participated in the research and filled out the questionnaire anonymously, with consent implied by submission. The protocol took about 30 minutes to be completed. All questions in the survey were set as mandatory, hence there were no missing answers. The data were then analyzed using IBM SPSS and RStudio. This study was conducted in accordance with the recommendations of the ethical code of the Italian Association of Psychology (AIP) and the ethical standards described in the 1964 Declaration of Helsinki. The materials and procedures used in this study were approved by the Ethical Committee for Scientific Research at the authors' institution (Ethics Committee Num. 36360).

### Data analysis

A hybrid Structural Equation Modeling (SEM), with BMI as an observed variable and dysmorphic concerns, eating disorders, and QoL as latent variables was used to examine a model in which BMI is considered the predictor variable, dysmorphic concerns the first mediator, eating disorders the second mediator, and QoL the outcome.

A parceling approach to obtain the indicators of the latent variables present in the current model was used, which consists of the aggregation of items (randomly selected) from the questionnaires in three indicators of each latent variable (75). Parcels are more likely to meet the assumptions of normality and less likely to be influenced by method effects (75, 76). The lavaan Package for R with the integration of RStudio were used to carry out the analysis of the covariance matrices and solutions were generated on the basis of maximum-likelihood estimation.

## Results

### Demographics and sample characteristics

Fifty nine percentage of the sample was on medication and median duration of PCOS diagnosis was 7.43 (5.69) years. Most of the individuals had medium-high educational qualifications. Specifically, 39% had a high school diploma, 56% had a university degree, and 2% had a post-graduate degree, whilst the remaining 3%, had a middle school certification. Concerning occupational status, 45% were employed, 14% were self-employed, 33% were students, 7% were unemployed, and 1% were housewives. With regard to marital status, 31% of individuals were single, 27% were engaged, 25% were living with a partner, 16% of individuals were married, and 1% were divorced. Furthermore, the participants reported having the following symptoms associated with PCOS: 88% had an irregular cycle, 75% had hirsutism, 58% were overweight or had difficulty maintaining a healthy weight, 55% had acne, 32% had infertility problems, and 21% had alopecia. Finally, concerning BMI, 57% had normal weight, 23% were overweight, 11% had obesity class I, 6% had obesity class II, and 3% had obesity class III (Table 1).

**Table 1:** Demographics and sample characteristics.

**Variables**	**Percentage (*N* subjects)**
**Educational qualification**	
Middle school certification	3% (12)
High school diploma	39% (172)
University degree	56% (242)
Post-graduate degree	2% (9)
***Occupational status***	
Student	33% (142)
Housewife	1% (7)
Unemployed	7% (30)
Employed	45% (196)
Self-employed	14% (60)
***Marital status***	
Single	31% (135)
Engaged	27% (117)
Cohabitating	25% (110)
Married	16% (72)
Divorced	1% (1)
***Bmi***	
Normal weight	57% (246)
Overweight	23% (101)
Obesity class I	11% (49)
Obesity class II	6% (28)
Obesity class III	3% (11)
***Pcos symptoms***	
Irregular cycle	88% (383)
Hirsutism	75% (327)
Overweight	58% (251)
Acne	55% (238)
Infertility	32% (138)
Alopecia	21% (91)

### Descriptive results and correlations

The values of skewness and kurtosis were examined to explore the distribution of the data (Table 2), suggesting that no problems were observed regarding the violation of the normal distribution. Furthermore, Table 2 shows the correlations among the dimensions of the questionnaires. Furthermore, Table 2 illustrates the correlations among the observed dimensions. BMI positively correlated with dysmorphic concerns and eating disorders, while negatively related with HRQoL. Furthermore, a positive association was found between dysmorphic concerns and eating disorders, while a negative correlation was observed between dysmorphic concerns and HRQoL. Finally, there was a negative correlation between eating disorders and HRQoL.

**Table 2:** Descriptive analysis and correlations.

	**α**	**M**	**SD**	**Skew**	**Kurt**	**1**	**2**	**3**
1. Body mass index	–	25.62	5.93	1.12	0.96	–	–	–
2. Dysmorphic concerns	0.93	3.04	0.95	−0.05	−0.89	0.37^*^	–	–
3. Eating disorders	0.88	19.72	12.89	0.72	−0.24	0.24^*^	0.65^*^	–
4. Quality of life	0.91	3.59	1.23	0.23	−0.43	−0.36^*^	−0.58^*^	−0.46^*^

N = 435;

* *p* < 0.01.

### Mediation model

**Figure 1:**
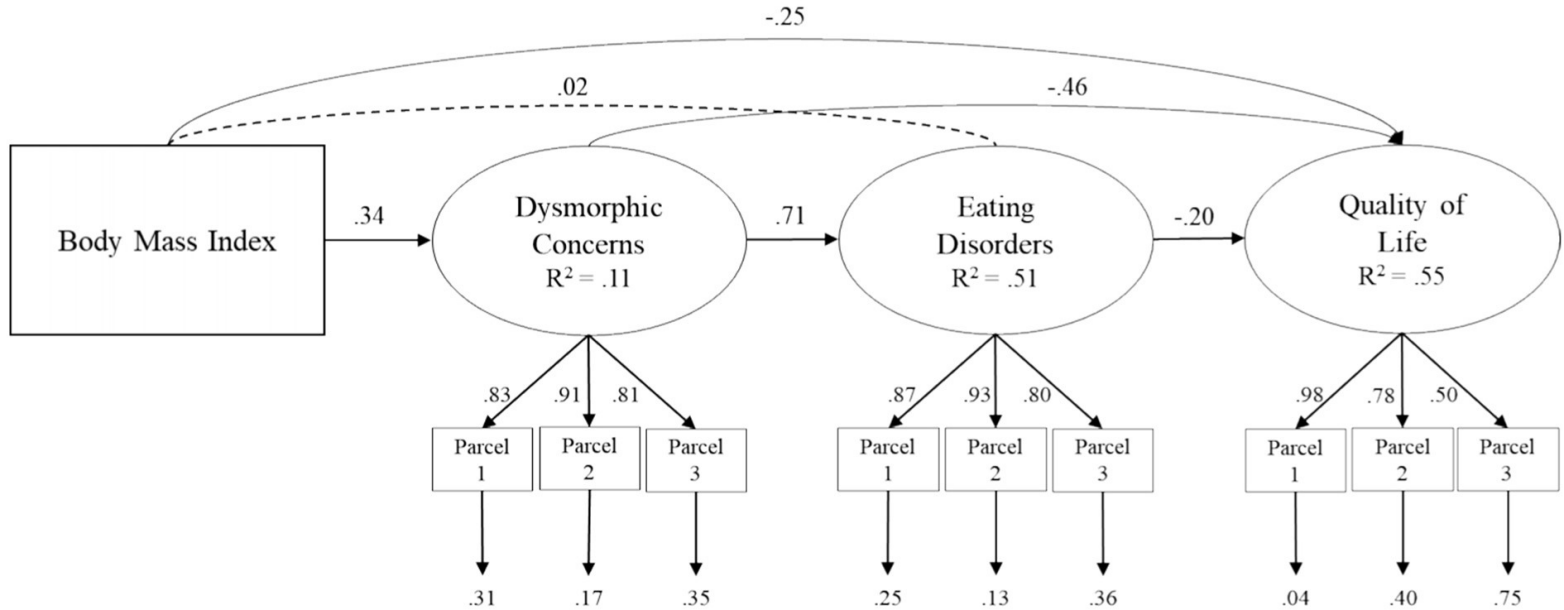
Structural model of association between body mass index, dysmorphic concerns, eating disorder and quality of life of life in individuals with PCOS. Coefficient shown are standardized path coefficients. Dotted lines represent not significance relations.

The model showed acceptable fit indices: χ2(30) = 167.93; *p* < 0.001, CFI =0.95, RMSEA =0.10 (90% CI =0.09 −0.12), SRMR =0.04 (Figure 1).

Significant paths were found from BMI to dysmorphic concerns (β = 0.34; *p* < 0.001) and HRQoL (β = −0.25; *p* < 0.001). Furthermore, the results showed a significant path from dysmorphic concerns to eating disorders (β = 0.71; *p* < 0.001) and HRQoL (β = −0.46; *p* < 0.001). Finally, eating disorders was related to HRQoL (β = −0.20; *p* < 0.001).

To explore the significance of the indirect effects that emerged (i.e., drop from the total to direct effect) we used the bootstrap-generated bias-corrected confidence interval approach (77, 78). A statistically significant indirect association was found from BMI to eating disorders by dysmorphic concerns (β = 0.24; *p* < 0.001) and from BMI to HRQoL by dysmorphic concerns (β = −0.15; *p* < 0.001). In addition, there was a significant indirect association from dysmorphic concerns to HRQoL by eating disorders (β = −0.14; *p* < 0.001). Finally, a statistically significant indirect relationships was found from BMI to HRQoL by dysmorphic concerns and eating disorders (β = −0.05; *p* = 0.002) (Table 3).

**Table 3:** Path estimates, SEs and 95% Cis.

	**β**	***p***	**SE**	**Lower bound (BC)**	**Upper bound (BC)**
				**95% CI**	**95% CI**
***Direct effect***					
BMI → dysmorphic concerns	0.34	< 0.001	0.07	0.03	0.05
BMI → eating disorders	0.02	0.67	0.00	−0.01	0.01
BMI → quality of Life	−0.25	< 0.001	0.01	−0.09	−0.04
Dysmorphic concerns → eating disorders	0.71	< 0.001	0.04	0.38	0.52
Dysmorphic concerns → quality of life	−0.46	< 0.001	0.14	−1.26	−0.73
Eating Disorders → quality of life	−0.20	< 0.001	0.19	−1.07	−0.32
***Indirect effect via dysmorphic concerns***					
BMI → eating disorders	0.24	< 0.001	0.01	0.01	0.02
BMI → quality of life	−0.15	< 0.001	0.01	−0.06	−0.03
***Indirect effect via eating disorders***					
BMI → quality of life	−0.01	0.69	0.00	−0.01	< 0.01
Dysmorphic concerns → quality of life	−0.14	< 0.001	0.09	−0.48	−0.14
***Indirect effect via dysmorphic concerns and eating disorders***					
BMI → quality of life	−0.05	0.002	0.00	−0.02	−0.01
					

SE, Standards errors; BC 95% CI, Bias Corrected-Confidence Interval; p, level of significance.

## Discussion

The current study tested a mediation model in which the relationship between BMI and QoL in individuals with PCOS is mediated by dysmorphic concerns, examining also the relations with eating disorders. In line with the expectations, the results showed that BMI was related to QoL not only directly but also indirectly through the mediation of dysmorphic concerns. In line with other studies supporting the relationship between body weight and body dissatisfaction (79, 80), the present findings corroborate the concept that excess body weight is one of the main factors contributing to impaired physical and mental functioning (81) and highlight how dissatisfaction with some aspect of one's own appearance contributes to shaping self-reported health status (82, 83). This may be due to the fact that a higher BMI fosters weight stigma and discrimination (84–86), which in turn may encourage social alienation and loneliness and thus decreased psychosocial adjustment. It may also be that weight problems due to PCOS negatively influence thoughts about attractiveness (87) and thus concerns about one's own potential appearance flaws are likely to occur.

Moreover, the present findings showed that individuals with PCOS reported lower QoL when dysmorphic concerns and eating disorders were higher because greater dysmorphic concerns involve fear of others' evaluations and constant approval-seeking, predisposing the person to a sense of incompetence and diminished social functioning (88–92). At the same time, given that eating disorder symptomatology impairs several dimensions of one's own functioning (47, 48), a lowered QoL is likely to occur. These results confirmed a plausible role for both variables in defining different levels of QoL and deepened their effects in the context of a burdensome condition such as PCOS. Furthermore, these findings support the hypothesis that BMI is connected to one's own QoL *via* the mediation of both dysmorphic concerns and eating disorders. Women with PCOS are more likely to have problems losing weight due to the metabolic features of the disease (93) and to have other comorbidities, including acne and hirsutism, which can cause dysmorphic concerns (39). These may foster maladaptive compensatory strategies used as a means of controlling food intake and body size, such as eating disorders. In the long run, this may alter individuals' functioning and result in impaired social, physical and mental adjustment. Moreover, the results of this study show that dysmorphic concerns, in addition to an indirect association with QoL by eating disorder symptomatology, have a direct link with this outcome dimension. Apprehensions of perceived defects in appearance may lead to increased dietary restriction and the use of compensatory strategies aimed at controlling body size and shape (73, 79, 94), contributing to an increased likelihood of disordered eating behaviors (79, 94). Consonant with previous findings revealing the direct link between body image dissatisfaction and disordered eating in women with PCOS (37, 57), the present study also found that dysmorphic concerns were related to eating disorder-related symptomatology. PCOS is often associated with a body shape that is culturally described as undesirable due to difficulties in weight management, which may lead individuals to undertake unhealthy dieting behaviors in an attempt to adhere to the idealized slim female figure, believed to be sexually attractive and healthy (63, 95, 96). In the long run, this may favor maladaptive eating habits used as a means for shaping one's own body (97). Overall, the results of the present study are consistent with the insights provided by Dokras et al. (98), who showed that health-related QoL scores are consistently reduced in PCOS, with difficulties in weight management playing a significant role in determining this condition. Interestingly, these results are consonant with recent advances that postulated from a biopsychosocial perspective that upregulated activation of the central HPG axis leading to PCOS may be epigenetically altered by abnormal eating patterns (55). Additionally, problematic body size, which typically denotes abnormal eating patterns, may hinder normal appetite signaling regulated by leptin and ghrelin, two important hormones controlling hunger and fullness (55). The present research has some limitations. First, it is a cross-sectional study, therefore future research could attempt to verify the findings and the direction of the hypothesized associations using a longitudinal design. Furthermore, the exclusive use of self-reports may introduce measurement bias and future studies may want to use a multi-informant approach to reduce this possibility. In addition, the research was conducted by reaching participants through social networks, so there may be problems with generalization in that only individuals with access to the Internet could be reached. It should be noted that the current results showed a higher number of women with problematic body sizes than those reported in a recent study based on the Italian population (99), which reported that the prevalence of obesity is about eight percent and 30 percent for overweight. PCOS often results in difficulties with weight management (7, 8), and this may partially explain the higher number of individuals with PCOS who reported overweight or obesity. Of note, the current study did not account for the different phenotypes of PCOS and focused only on the presence of dysmorphic concerns and eating disorder symptoms in this clinical population. Future studies should replicate this model for different PCOS phenotypes in order to provide a more nuanced description of the disease. Furthermore, although our study reported the number of participants currently on medication, it did not take into account important factors such as the type of medication used and adherence to the treatment plan. Future studies are needed in which the influence of drug treatment on individual quality of life is also observed. However, our study advances the current state of knowledge by explaining the relationships between BMI and QoL in individuals with PCOS. It would be thought-provoking to implement future studies in which a retrospective approach is used to observe the potential influence of the COVID-19 pandemic scenario (100, 101) because recent findings observed that during isolation due to the COVID-19 outbreak, weight gain was slightly more pronounced in individuals suffering from PCOS and this may be due to impaired sleep quality and eating habits rather than reduced physical activity (102). In a similar vein, future studies may assess how different eating patterns ad caloric restriction protocols like ketogenic diet (103, 104) may interact with individuals' perception of the condition of their own health. Similarly, future studies may benefit from the implementation of other biological variables, such as energy expenditure and fat mass (105), as they may influence adaptations of metabolism and body composition. More importantly, this study contributes to the evidence by investigating the role that dysmorphic concerns and eating disorder symptomatology may have in explaining the individual differences in the association between BMI and QoL in individuals with PCOS. In light of the findings of this study, there may be important clinical and research implications. This study highlights how people with PCOS and problematic weight have a poorer QoL. Individuals suffering from PCOS would benefit from a multidisciplinary intervention aimed at managing weight and dealing with problematic thoughts concerning appearance and weight. In addition, our results show how both dysmorphic concerns and eating disorders are relevant in the context of PCOS. Individuals with PCOS may benefit from periodic assessments aimed at detecting disordered eating (98). Practitioners should therefore screen individuals with the condition for a problematic relationship with food or excessive concerns about their bodies. Intervention and prevention programmes may also want to include women's partners in order to improve their effectiveness, as the disease may negatively affect women's intimate relationships (67). From a public health perspective, these results emphasize the detrimental effects of dysmorphic concerns and eating pathology on PCOS patients and require the urgent implementation of health promotion interventions. Policy makers and health institutions should thus create health promotion interventions aimed at improving knowledge, communication, and beliefs about the impact of PCOS on quality of life. This is consonant with recent advances arguing that mental health literacy may help promote individuals' psychosocial functioning (106). In addition, these programmes should not only involve PCOS patients, but also socialization agents such as family, peers, and the media through the transmission of messages aimed at raising awareness about appearance-related pressures that may come from social sources to prevent the onset of unhealthy body image (28). Finally, this study provided a holistic approach to the understanding of complex conditions such as PCOS.

## Data availability statement

The data that support the findings of this study are available from the corresponding author.

## Ethics statement

The studies involving human participants were reviewed and approved by Ethics Committee of the Center for Research and Psychological Intervention of the University of Messina. The patients/participants provided their written informed consent to participate in this study.

## Author contributions

NB assisted with manuscript preparation, study design, data analysis and interpretation and data were gathered on-line under her supervision. DC assisted with manuscript preparation and data interpretation. MC assisted with manuscript preparation and data interpretation and served as corresponding author. VV assisted with concept and study supervision. All authors contributed to the article and approved the submitted version.

## Conflict of interest

The authors declare that the research was conducted in the absence of any commercial or financial relationships that could be construed as a potential conflict of interest.

## Publisher's note

All claims expressed in this article are solely those of the authors and do not necessarily represent those of their affiliated organizations, or those of the publisher, the editors and the reviewers. Any product that may be evaluated in this article, or claim that may be made by its manufacturer, is not guaranteed or endorsed by the publisher.
